# Creation of Chemically Tri-Layered Collagen Crosslinked Membranes and Their Comparison with Ionically Tri-Layered Chitosan Crosslinked Membranes to Study Human Skin Properties

**DOI:** 10.3390/ijms241713443

**Published:** 2023-08-30

**Authors:** Larry Galán-Navea, Rocío Guerle-Cavero, Albert Balfagón-Costa, Beatriz Artalejo-Ortega

**Affiliations:** Pharmaceutical Chemistry Research Group, Institut Químic de Sarrià, Universitat Ramón Llull, 08017 Barcelona, Spain; larrygalann@iqs-blanquerna.url.edu (L.G.-N.); rocioguerlec@iqs.url.edu (R.G.-C.); beatriz.artalejo@iqs.url.edu (B.A.-O.)

**Keywords:** collagen, chitosan, human skin, crosslinking, membrane, layers, elasticity, rheology

## Abstract

In 2009, a new European regulation came into force that forbade the use of animals in the cosmetics industry. As a result, new alternatives were sought, taking into account the new ethical considerations. The main objective of this article is to continue a line of research that aims to build a physical model of skin from a biomaterial scaffold composed of collagen, chitosan or a combination to investigate whether they offer similar behavior to human skin. Collagen, the major component in the dermis, was crosslinked with glutaraldehyde (GTA) to develop three formulations for studying some properties of the skin through rheological tests like swelling index, elasticity or water loss. In addition, this article makes a comparison with the results obtained in the previous article where the membranes were made of chitosan and tripolyphosphate (TPP). The results obtained highlight that the tri-layered membranes scaffold better than the mono-layered ones to increase the elastic modulus (G′) and the permeability. Furthermore, they offer a protective effect against water loss compared to mono-layered membranes. As regards chitosan membranes, these have a higher G′ modulus than collagen membranes when the degree of deacetylation (DDA) is 85%. However, collagen membranes are more elastic when the DDA of chitosan is 76%, and their linear viscoelastic limit (LVL) doubles that of chitosan membranes, both for the degree of acetylation of 76 and 85%.

## 1. Introduction

Due to technology and scientific advances that have been developed over the years, life expectancy has lengthened considerably, and human quality of life has improved greatly. Currently, people try to take care of themselves in all aspects (internally and externally), and for this reason, the pharmaceutical and cosmetic industry has developed multiple formulations and cosmetic products to satisfy these needs, which are increasingly demanded by different population groups (age, sex, culture, etc.). These products have increased enormously, not only in their claims but also in their formulations, which have been developed to cover all types of problems, including anti-ageing creams, sun milks, facial cleansing foams, moisturizing lotions, etc. However, all these products are formulated with substances and components that can have negative effects, such as skin and eye irritation or other allergic reactions. Since people use these products on a daily basis for personal hygiene and skin health, their quality and safety must be guaranteed through a variety of studies. In the past, these studies were carried out by testing them on animals, but since 30 November 2009, the European Parliament and the Council of the European Union published Regulation 1223/2009 on cosmetic products in which it was prohibited. Specifically, in Chapter V, Article 18, it prohibits the sale in the European Union of any cosmetic product that has been tested on animals and also extends this to cosmetics whose individual ingredients or mixtures of ingredients have been tested on animals [1].

To assess the efficacy of cosmetic and dermo-cosmetic products, several validated test methods are available. Initially, most of these test methods involved the use of animals, but there have been many advances to eliminate their use. In addition, EU regulation 2010/63/EC on the protection of animals for scientific purposes requires and encourages the use of alternatives to animals, such as in vitro skin models using collagen membranes that simulate human skin [2]. These in vitro models are used to perform safety and toxicity testing of cosmetics and ingredients, as well as to evaluate efficacy, support product claims and confirm product composition [3].

The European Centre for the Validation of Alternative Methods (ECVAM) [3] suggested multiple in vitro alternatives, among which emerged skin models using prototypes produced with epidermal keratinocytes that have been reconstituted in the human epidermis known commercially as EpiSkin^TM^ (L’Oreal, Île-de-France, France) and EpiDerm^TM^ Skin Corrosivity Test (MatTek Life Science; Ashland, MA, USA), among others, which allow distinguishing between corrosive and non-corrosive substances by simulating the real behavior of human skin [3,4]. However, the development of these methods is complex and costly, requiring the application of physicochemical, biochemical and cell culture principles. Therefore, choosing biomaterials for this requires that they have similar characteristics to the tissue being simulated. As type I collagen is the most prevalent protein in the body and one of the main components of the extracellular matrix (ECM) [5], this work proposes the first stage in the development of biocompatible type I collagen membranes. In addition, another reason for choosing marine collagen has been due to ethical constraints related to avoiding porcine and bovine products [6]. As they can transmit bovine spongiform encephalopathy (BSE) and transmissible spongiform encephalopathy (TSE), the industry considers the marine collagen source an important alternative. Marine collagens have the advantages of high yield and no risk of disease transmission that can be obtained from marine invertebrate animals or fish. Mammalian collagens have a higher thermal stability due to a higher content of amino acids compared to marine collagens [7], as marine collagens have a low denaturation temperature. The mechanical strength of marine collagen is poorer than bovine extracted collagen because it is less crosslinked. After crosslinking treatment, marine collagen can be used as a biomaterial in tissue engineering [8,9]. Despite some limitations, marine collagen is an attractive option for pharmaceutical and biomedical applications [10] because the source is cheap and there is no risk of BSE or TSE [11].

The characteristics of collagen fibers increase the covalent bonds between the tropocollagen chains through crosslinking, which increases the tensile strength of the polymeric network [12,13]. On the other hand, once the type I collagen membrane simulating skin layers was obtained, it was characterized to determine its rheological properties [14]. The membrane was obtained by chemical crosslinking with glutaraldehyde [15]. Crosslinking is a covalent reaction involving two closely related protein amino acid residues. Usually, the two functional groups of the amino acid side chains occur, resulting in an intermolecular or intramolecular bond [16]. As the polymer is crosslinked, it becomes harder and more compact compared to when it is uncrosslinked. As will be shown in this study, this technique favors the elasticity between membrane layers, especially when the membranes consist of three layers, where the modulus of G′ is greatly increased. In addition, this fact is in common with the results of the previous article, where the tri-layered chitosan membranes had a higher G′ modulus than the mono-layered ones [17].

Glutaraldehyde is a di aldehyde whose aldehyde groups are highly reactive and can form covalent bonds with functional groups such as amines [18], reacting with α-amino groups of amino acids [19], which in this case are present in the protein structure of collagen. It is the most popular crosslinking agent because it is very effective in stabilizing biomaterials, is accessible and affordable. GTA is a non-zero crosslinker, in other words, a part of its molecule is incorporated into that of the formed protein structure [20]. In addition, chemical crosslinkers, unlike physical crosslinkers, generate covalent bonds that increase the mechanical properties of the generated scaffolds such as flexibility and tensile strength [21,22,23,24].

The skin can suffer from alterations such as dehydration, ageing, hyperpigmentation, etc. These factors can disrupt the intrinsic properties of the skin. Elasticity can vary according to gender, location on the body, age, body mass index and skin thickness. In order to have a reference of the actual skin elasticity values and then to compare them with those obtained in this study, the values for different body locations and classified by sex are presented [25] (Table 1):

There are some novelties in this study in relation to older collagen studies. One of them is creating a three-layer collagen membrane with a procedure that is explained in detail, as well as detailing the development of three different formulations by means of a statistical study (Design of Experiment). Another novelty is performing the curing process on the membranes to increase their elasticity and demonstrating this numerically. Finally, the effect on permeation with mechanically made pores has been studied. With respect to the previous articles [17,26], the type of water present in collagen membranes has been studied by means of the weight loss test. On the other hand, properties such as elasticity or swelling index have been differentiated between each other in order to widen the range of possibilities for future use. This article proposes to broaden the range of properties of this type of biomaterial according to the application to be carried out with them.

## 2. Results and Discussion

All tests were carried out with membranes die-cut to a diameter of 2 cm. For both mono-layer and tri-layer membranes, their thickness and weight were recorded, see Table 2.

As can be seen, the tri-layered membranes are somewhat heavier and slightly thicker than the mono-layered membranes. This may be due to the amount of water retained in the membranes and the capacity they have to release this water when evaporating. This will affect the results of the characterization tests that will be evaluated below.

### 2.1. Rheological Study

**Figure 1:**
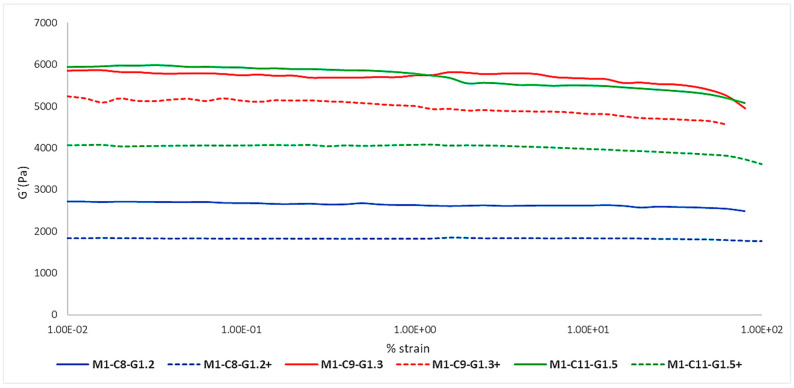
Impact of the effect of evaporation on the elastic modulus of mono-layered membranes.

**Figure 2:**
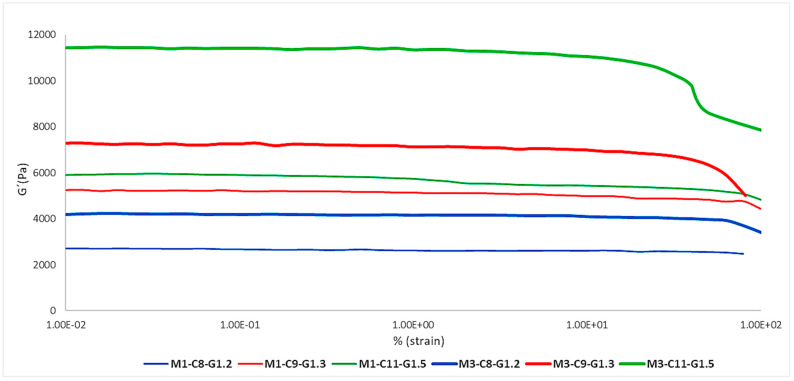
Impact of the effect of the number of layers on the elastic modulus (G′).

**Figure 3:**
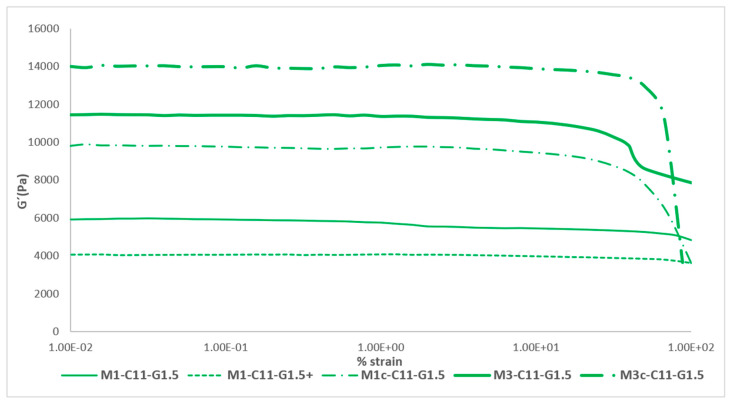
Representation of the set of effects on the elastic modulus of the best formulation, Mx-C11-G1.5.

The rheological test is the test that guided the way to obtain the desired membranes. For this purpose, different variables that could affect the characteristics of the membranes manufactured (modulus G′) were evaluated, such as the number of layers, evaporation time, % *w*/*w* of each formulation, curing effect and thickness. In Figure 1, Figure 2 and Figure 3 and Figure S1–S3 the abscissa axis (x) represents the % strain or deformation, and the ordinate axis (y) represents the elastic modulus or G′. For each of the rheological tests, two repetitions were carried out, so the tables with the mean of the G′ modulus are included with their respective standard deviation (SD). In order to facilitate the interpretation of the graphs, a membrane identification system has been designed for the graphs belonging to the rheology assay (Table 3).

**Table 3:** Identification system using colors and patterns for each kind of membrane.

Formulation		Mono-Layered Membrane; 1 Day	Mono-Layered Membrane; 2 Days (+)	Tri-Layered Membrane; 1 Day	Cured Mono-Layered Membrane; 1 Day	Cured tri-Layered Membrane; 1 Day
Mx-C8-1.2		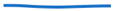	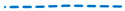	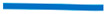	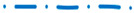	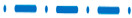
Mx-C9-1.3		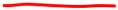	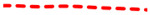	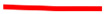	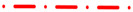	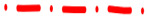
Mx-C11-1.5		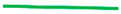	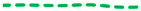	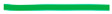	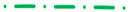	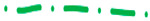

In addition, a table with the mean of G′ modulus and its SD has been included in each graph to enhance the comparison between membranes (Table 4, Table 5, Table 6, Table 7, Table 8 and Table 9).

#### 2.1.1. Evaporation Effect 

As can be seen in Table 3, the same formulation had two different resting times from when the membrane was made to when the test was carried out, with 1 day being the shortest time, represented by the continuous lines, and 2 days being the maximum time, represented by the dashed lines (Figure 1). 

**Table 4:** Mean and standard deviation (SD) of the G′ modulus of each membrane to study the effect of evaporation.

Membrane ID	Mean G′ (Pa) ± SD
M1-C8-G1.2	2638.95 ± 216.54
M3-C8-G1.2+	1822.29 ± 104.76
M1-C9-G1.3	5685.29 ± 284.25
M3-C9-G1.3+	5088.73 ± 267.44
M1-C11-G1.5	5695.77 ± 192.85
M3-C11-G1.5+	4003.29 ± 96.55

The main information transmitted by the graph in Figure 1 is that all formulations share the same premise: the most elastic membrane was the one with the shortest evaporation time, 1 day. As can be seen, the continuous lines represent higher values of elasticity (G′) than the dashed lines for the three formulations. 

This may be due to the tension that resides in the internal structure of the membranes once they were formed. Over the course of the days, they relax and lose their firmness, also causing a considerable reduction in their elasticity, in other words, they become more plastic. At the same time, they suffer from the effect of evaporation, so that the loss of water directly affects their flexibility.

#### 2.1.2. Layering Effect

Figure 2 shows the effect of having one or three layers present in the membrane on the elasticity of the different formulations. As can be seen, each formulation was within a different range of G′ modulus values, with the most elastic formulation being Mx-C11-G1.5 (green) and the least elastic being Mx-C8-G1.2 (blue). On the other hand, there was an increase in elasticity in the tri-layered membranes (thick lines) and this was common to all the formulations, so it can be confirmed that it is proportional.

**Table 5:** Mean and standard deviation (SD) of the G′ modulus of each membrane to study the effect of the layers.

Membrane ID	Mean G′ (Pa) ± SD
M1-C8-G1.2	2638.95 ± 216.54
M1-C9-G1.3	5685.29 ± 284.25
M1-C11-G1.5	5695.77 ± 92.85
M3-C8-G1.2	4114.66 ± 559.36
M3-C9-G1.3	7014.64 ± 196.32
M3-C11-G1.5	11,012.53 ± 100.60

#### 2.1.3. Effect of Curation Process

In this case, as can be seen (Figure S1), each of the formulations was again differentiated in their elasticity ranges. The cured tri-layered membranes increased the elasticity even more than the cured mono-layered membranes, so their effect was beneficial for the membrane. Furthermore, an increase in the curing effect was seen as the % *w*/*w* of both collagen and GTA increased, so the G′ interval between the cured mono-layered and tri-layered membrane of the formulations became larger as the concentration increased. Therefore, it can be confirmed that the effect of curing is synergistic to the concentration of its components, since it increases the crosslinking of the groups that have not reacted during the initial membrane creation process. In addition, if the results of the chitosan membranes article [17] are compared, there are similarities in the behavior of both materials in the curing process in the case of chitosan membranes with non-crosslinked layers. Both chitosan and collagen produced an increase in the elastic modulus of the membranes when they are subjected to this process. In the case of collagen with the Mx-C11-G1.5 formulation, the elastic modulus increased around 3 kPa comparing with the tri-layered non-cured membrane, and for chitosan with an 85% DDA, it increased around 22 kPa. 

In this case, in both figures (Figures S2 and S3), it can be confirmed that the curing effect of the cured membranes produced an increase in elasticity with respect to the same membranes that had not been subjected to the curing process.

**Table 6:** Mean and standard deviation (SD) of the G′ modulus of each membrane to study the effect of curing process in tri-layered and mono-layered membranes.

Membrane ID	Mean G′ (Pa) ± SD
M1c-C8-G1.2	3946.16 ± 373.22
M1c-C9-G1.3	5758.55 ± 157.37
M1c-C11-G1.5	9329.93 ± 417.36
M3c-C8-G1.2	5246.19 ± 202.30
M3c-C9-G1.3	8607.78 ± 95.96
M3c-C11-G1.5	13,677.95 ± 281.44

**Table 7:** Mean and standard deviation (SD) of the G′ modulus of each membrane to study the effect of the curing process in tri-layered cured and non-cured membranes.

Membrane ID	Mean G′ (Pa) ± SD
M3c-C8-G1.2	5246.19 ± 202.30
M3c-C9-G1.3	8607.78 ± 95.96
M3c-C11-G1.5	13,677.95 ± 281.44
M3-C8-G1.2	4114.66 ± 559.36
M3-C9-G1.3	7014.64 ± 196.32
M3-C11-G1.5	11,012.53 ± 100.60

**Table 8:** Mean and standard deviation (SD) of the G′ modulus of each membrane to study the effect of the curing process in mono-layered cured and mono-layered non-cured membranes.

Membrane ID	Mean G′ (Pa) ± SD
M1c-C8-G1.2	3946.16 ± 373.22
M1c-C9-G1.3	5758.55 ± 157.37
M1c-C11-G1.5	9329.93 ± 417.36
M1-C8-G1.2	2638.95 ± 216.54
M1-C9-G1.3	5685.29 ± 284.25
M1-C11-G1.5	5695.77 ± 92.85

#### 2.1.4. Set of Effects

Finally, Figure 3 shows all the effects studied for the Mx-C11-G1.5 formulation in the rheological test and their effects on the elasticity of the membrane (these results can be compared with the previous scientific literature [24,27]). As can be seen, there was an evolution of the membrane towards the objective that was set, that of obtaining a membrane with rheological characteristics similar to those of the skin. 

The maximum value of elasticity that could be reached during the study was around 14 kPa and was obtained with the cured tri-layered membrane. This value was comparable to the elasticity that can be obtained in a forearm, a chest wall or an abdominal wall (Table 1). These values are close to reality and were one of the objectives set at the beginning of the work. On the other hand, it can be seen how the linear visco-elastic limit (LVL) decreases as the complexity of the membrane increases, so it can be stated that they are inversely proportional. The LVL is the maximum deformation value that the membrane can be subjected to while maintaining its intrinsic elasticity. From this maximum deformation limited by the LVL, the elasticity values decrease considerably.

**Table 9:** Mean and standard deviation (SD) of the G′ modulus for Mx-C11-G1.5 with all the effects.

Membrane ID	Mean G′ (Pa) ± SD
M1-C11-G1.5	5695.77 ± 192.85
M1-C11-G1.5+	4003.29 ± 96.55
M1c-C11-G1.5	9329.93 ± 417.36
M3-C11-G1.5	11,012.53 ± 100.60
M3c-C11-G1.5	13,677.95 ± 281.44

If the results obtained from these membranes are compared with those of the article on chitosan [17], significant differences can be seen. On the one hand, as reported in the previous article, chitosan membranes had a much higher elastic modulus than collagen membranes, even reaching 54 kPa among the best membranes obtained. However, if chitosan membranes with a DDA of 76% are compared, it can be seen that all their G′ modulus were lower than collagen membranes, with the best tri-layered membrane obtaining a G′ of 4 kPa or even 5.4 kPa for a membrane with pores compared to a collagen tri-layered membrane obtaining a G′of 14 kPa. On the other hand, chitosan seems to offer a disadvantage compared to collagen, as the LVL of its membranes is much lower, even less than half. While collagen membranes last around 10 min, chitosan membranes start to decay after 3–3.5 min. This directly expresses that the resistance of collagen membranes to mechanical forces is twice as good as that of chitosan membranes.

### 2.2. Water Loss Assay

Water loss in membranes can be classified into two types: free water, which is water that can evaporate, and fixed water, which is intrinsic to the material itself and therefore cannot be eliminated. In this case, the aim of this test was precisely this, to see what type of water the membranes had and to compare it with the % *w*/*w* H_2_O in each of the formulations. In addition, as will be seen below, the membranes exert a “protection” against this water loss, which will also be evaluated.

**Figure 4:**
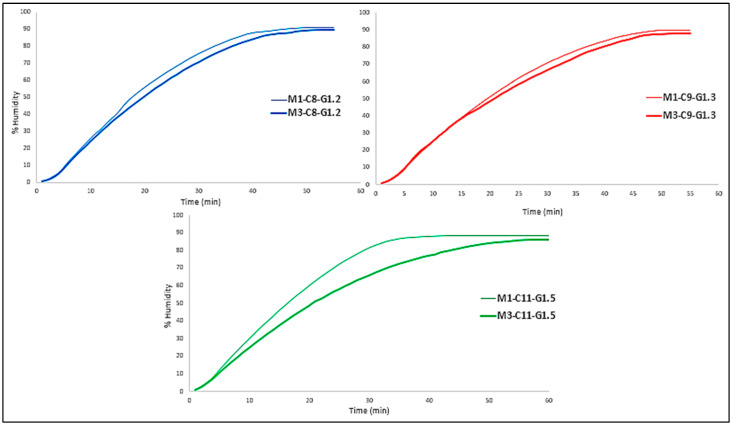
Comparison of water loss of mono- and tri-layered membranes for each formulation.

As can be seen in Figure 4, in all three cases the tri-layered membrane provides slightly more protection against water loss than the mono-layered membrane. This may be due to the fact that both the bottom and top layers give the middle layer greater protection by being sandwiched between them, giving it a shielding effect.

The fact that the formulations change and there are variations in % *w*/*w* resulting in more or less crosslinking does not seem to affect this aspect, as there is no significant difference in water loss between formulations, they are quite similar.

**Figure 5:**
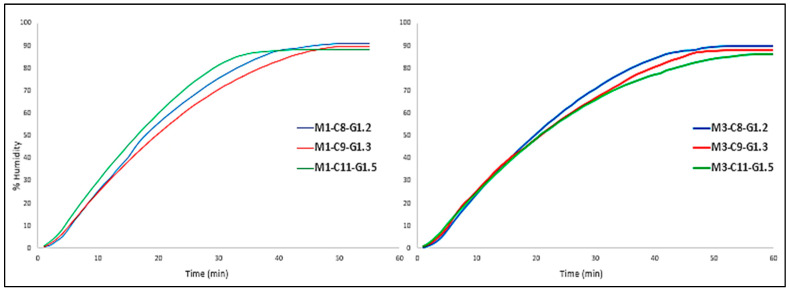
Comparison of water loss of mono-layered membranes (**left**) and tri-layered membranes (**right**) for different formulations.

However, if the three formulations with the same number of layers are compared, a conclusion can be drawn (Figure 5).

In this case, the formulations behave quite similarly, regardless of the number of layers; that is to say, with respect to the final time of the assay (60 min), Mx-C8-G1.2 formulation (blue color) loses the most water and Mx-C11-G1.5 (green color) the least. 

This fact is given by the formulation itself, as the more water it has, the more water it is likely to lose, and in this case this is true.

Mx-C8-G1.2 → 8% Collagen + 1.2% GTA = 9.2% → 90.8% H_2_O.

Mx-C9-G1.3 → 9% Collagen + 1.3% GTA = 1.3% → 89.7% H_2_O.

Mx-C11-G1.5 → 11% Collagen + 1.5% GTA= 12.5% → 87.5% H_2_O.

If the theoretical values are compared with the experimental values shown in Table 10, it can be clearly seen that each formulation is very close to the theoretical values of % *w*/*w* of water it contains. In addition, the previously mentioned shielding effect can be seen. For example, for formulation Mx-C11-G1.5, the theoretical water value was 87.5% *w*/*w*. Experimental values showed that indeed both the mono (88.22%) and tri-layered (86.03%) membranes were close to the theoretical value. 

However, the % of water in the tri-layered membrane was lower than both the mono-layered and the theoretical one, as it protects against this water loss. On the other hand, the experimental value for the single membrane was slightly higher than the theoretical value, which may indicate that the solutions were not 100% homogeneous due to the high viscosity, and this could lead to these variations in the % water. 

Finally, comparing the theoretical values with the experimental ones, it can be determined that most of the water present in this type of collagen-scaffolded polymers was free water, since the % *w*/*w* of water in the formulation was practically the same as the % by weight of water lost by the membrane.

### 2.3. Swelling Index

**Figure 6:**
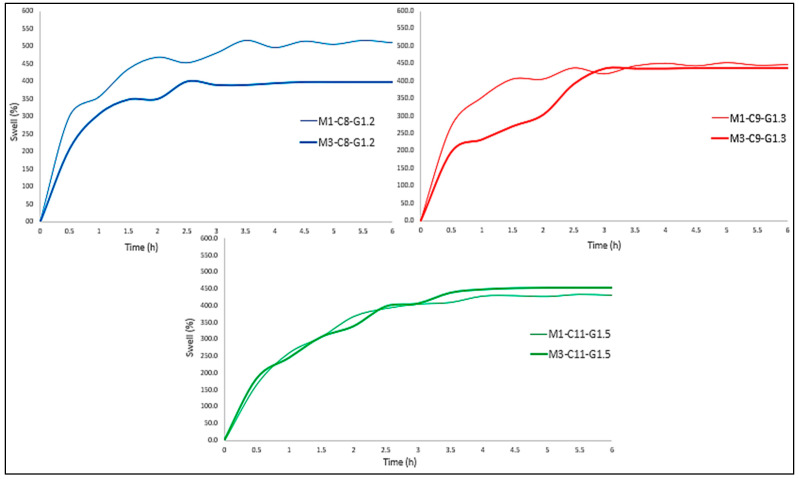
Comparison of the swelling index of mono- and tri-layered membranes for each formulation.

As detailed throughout this work, the membranes are intended to be used in in vitro tests for the development of dermo-cosmetic formulations, so they will be subjected to products that will have to be absorbed, so it is essential to evaluate this capacity. The absorption capacity of each of the formulations is shown in Figure 6, comparing the mono-layered and tri-layered membranes (these results can be compared with previous scientific literature [28]).

**Figure 7:**
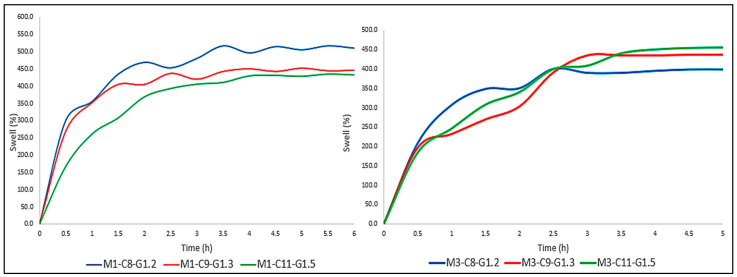
Comparison of the swelling index of mono-layered membranes (**left**) and tri-layered membranes (**right**) for each formulation.

As can be seen in Figure 6, the period of greatest absorption is the first 30–45 min, which was common to all membranes. On the other hand, the tri-layered membranes stabilized after 150–180 min. In other words, they maintained a constant weight and stopped expelling and absorbing PBS. Mono-layered membranes, on the other hand, responded in a completely different way, maintaining a somewhat less stable behavior throughout the 6 h of the test (Figure 7).

Comparing the initial weights with the final weights of each of the membranes, it can be seen that the Mx-C11-G1.5 formulation was the only one that moderately maintained the weight of the membrane. In other words, these membranes had the capacity to lose all the water present in their internal structure and subsequently absorb up to 72% of their dry weight (Table 11).

As can be seen, as the concentration of the formulation increased, this capacity also increased, so it was possible that this is the result of cause–effect. However, if the behavior is compared with chitosan membranes, it can be seen that the highest absorption does indeed occur during the first half hour, but the absorption capacity of collagen membranes was higher than chitosan membranes. In the case of chitosan 85% DDA, a maximum swelling of 107% ± 7.4% and 114% ± 5.2% was obtained, for both supplier origins studied and 256% ± 63.6% for chitosan of 76% DDA [17]. 

In addition, the stability offered by chitosan was greater than that of collagen, since after the first hour, chitosan tri-layered membranes were completely stabilized, whereas collagen membranes did not reach a constant weight until 3 h later.

### 2.4. Permeation Assay

This test was performed 24 h after the membranes were produced. After this time, the membranes were fixed between two permeation rings placed between the receiving and donor compartments in the Franz cell. Then, 30 mL of water was introduced into the donor compartment and remained stagnant. As time elapsed, the water permeating through the membrane into the receiving compartment was collected with the aid of a Pasteur pipette using the sampling arm. This water was collected in a pre-weighed beaker, and the amount obtained was quantified. 

**Figure 8:**
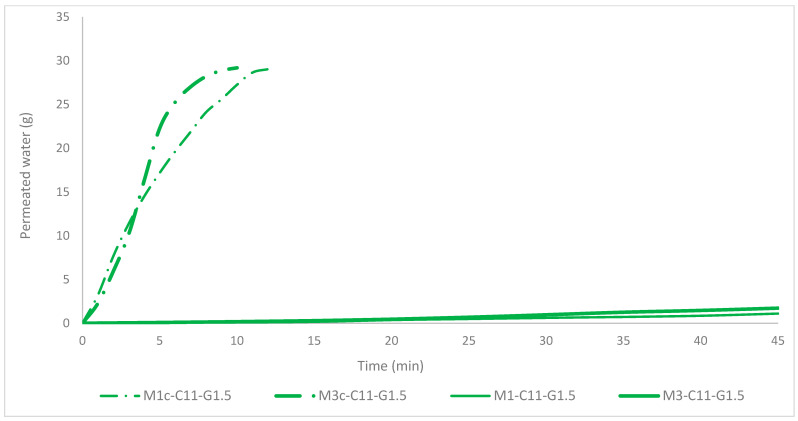
Comparison of the performance of the Mx-C11-G1.5 formulation for water permeation through different membranes.

Clearly, two very distinct tendencies can be observed that indicate whether the membrane had been activated by the DermaStamp^®^ or not (Figure 8). On the one hand, the membranes that were perforated (M1c-C11-G1.5 and M3c-C11-G1.5) were permeated by all the water in 10–12 min, while on the other hand, those that were not activated (M1-C11-G1.5 and M3-C11-G1.5) were permeated by about 2% of the water all throughout the 45 min test (results can be compared with previous literature [29]).

Comparing these results with those of the chitosan article, the permeation behavior of the membranes is quite similar, also differentiating between those that are perforated and those that are not with two very distinct trends. However, the time varies considerably compared to the chitosan membranes, since the time for permeation of 30 g of water in chitosan 85% DDA was 2.5–2.9 min, in the case of collagen it is 10–12 min. The pores appear to be much smaller for collagen-based membranes [17]. This fact demonstrates the need to create pores if the membrane needs to be permeable or, on the contrary, to dispense with this feature by leaving the membrane deactivated. 

### 2.5. Scanning Electron Microscopy

To determine the surface morphology of the membranes, it was decided to observe under the microscope both a mono-layered membrane and a tri-layered one of the Mx-C11-G1.5 formulation, as this was the one with which the best results were obtained throughout the study. 

**Figure 9:**
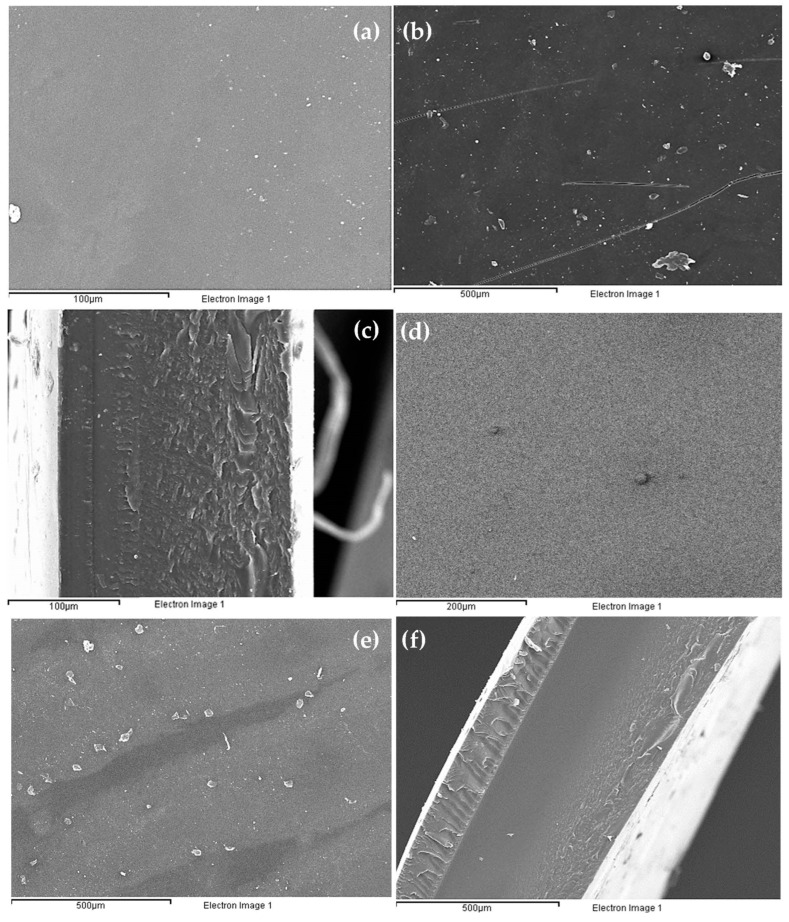
SEM micrographs of formulation Mx-C11-G1.5: (**a**) bottom view of M1-C11-G1.5; (**b**) top view of M1-C11-G1.5; (**c**) profile view of M1-C11-G1.5; (**d**) bottom view of M3-C11-G1.5; (**e**) top view of M3-C11-G1.5; (**f**) profile view of M3-C11-G1.5.

For the bottom view images (a and d) in Figure 9, it can be seen that the surface morphology was more uniform than the top view (b and e) for both membranes. This was because the bottom side had been in contact with the Petri dish from the time the membrane was formed until it was observed under the microscope, and therefore, it could not have been disturbed by the environment. In addition, the Petri dish was a completely smooth surface whereas the outermost part of the membrane had been in constant contact with air, hence the particles observed.

**Figure 10:**
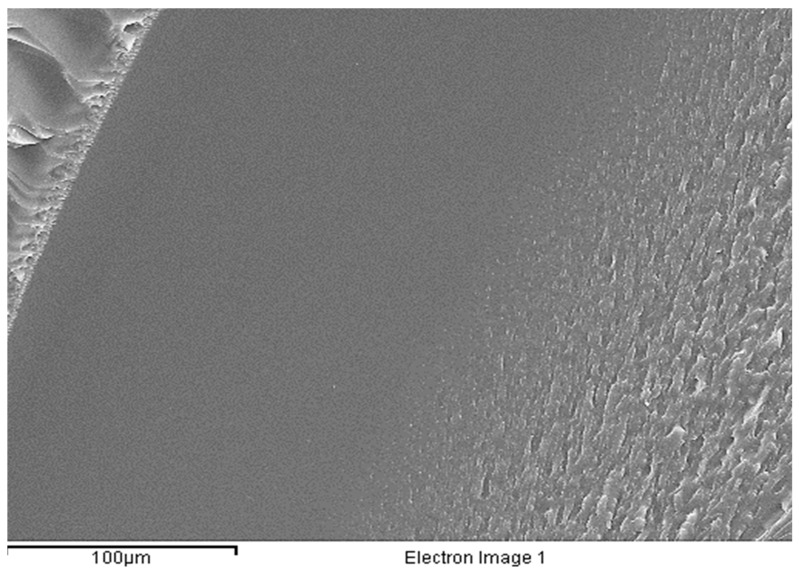
SEM micrograph of the differentiation of the three layers for the M3-C11-1.5 membrane.

On the other hand, a clear differentiation in the number of layers can be seen in both the mono- (c) and the tri-layered (f) membranes. This distinction in the tri-layered membrane may be due, apart from the formation of the three layers distinguished by the methodology, to the fact that the middle layer had not been in contact with air while the upper one had been. The lower layer was also the base layer, and as explained above, it had a higher grammage than the other two. These three factors may have been the factors that led to a fully specialized tri-layered membrane (Figure 10).

## 3. Materials and Methods

### 3.1. Materials

Gelatinized cold water fish skin collagen powder, 40–50% in H_2_O, (product batch G7041-500G) and Glutaraldehyde 50 wt. % in H_2_O were obtained from Sigma-Aldrich^®^ (St. Louis, MO, USA) (product code (340855-1L). The phosphate-buffered saline tablet was supplied by Sigma, Merck Life Science S.L. (Madrid, Spain) (SLCD5938). The phosphate-buffered saline tablet dissolved in 200 mL deionized water yields 0.01 M phosphate buffer, 0.0027 M potassium chloride and 0.137 M sodium chloride, pH 7.4, at 25 °C. Extra pure anhydrous calcium chloride granules, 93.0%, were used from Scharlab S.L (Catalonia, Spain) (product code 17500501). Qualitative analysis filter paper 90 mm was obtained from Filter-LAB^®^ (6013) (Catalonia, Spain). The balances were an AND Weighing (Stratford-Upon-Avon, UK) EK-300i and a COBOS Precision (Catalonia, Spain) CS-300M. The microneedle device for pore making was a 140 DRS DermaStamp^®^ system purchased through GBS International holding Ltd. (Beijing, China). It consisted of 140 stainless steel microneedles; the needle width was 0.25 mm and the needle spacing was 1.58 mm. The depth of the needles could be adjusted from 0.2 to 3.0 mm, however in this work was set to 3.0 mm to ensure that they drill all layers of the membrane. The Franz cell was from Fisher Scientific S.A. (Hudson, NH, USA) and manufactured by Fisher Scientific, S.L. (Madrid, Spain). Petri dishes were obtained from Thermo Fisher Scientific^®^ (Warrington, UK).

### 3.2. Methods

All experiments were carried out at a temperature between 19 and 23 °C and a relative humidity between 65 and 85%.

#### 3.2.1. Obtaining the Formulations

To obtain the different membrane formulations, 3 trifactorial 2-level Design of Experiments (DOE) were carried out, experimentally evaluating two variables: the elastic modulus (G′) and the drying time of the membrane from its creation to its characterization in the rheometer. (Table 12). 

In addition, in order to draw a conclusion from each DOE and perform the next one, the effect of each of the variables and their interactions with each other was evaluated from the G′ values of each of the membranes (Tables S6–S14).

Finally, taking into account the G′ modulus, the best formulation of each DOE was selected (Table 13) to carry out all the subsequent characterization tests and thus obtain a much broader and more representative comparative study. 

#### 3.2.2. Preparation of the Collagen Solution

For the collagen solution preparation, the first step was to weigh the required amount of collagen into a 50 mL beaker. Subsequently, 20.0 mL of deionized water was poured over the previously weighed collagen powder. The mixture was then stirred constantly for 45–50 min using a magnetic stirring rod at 300 rpm and heated to a temperature of 60 °C. 

It is critical not to stir too much as air bubbles would be incorporated into the mixture which would be very difficult to remove and which would of course affect both the formation of the membrane and its integrity for subsequent characterization. 

After 40–45 min the collagen should be completely dissolved. The solution was then separated from the hot plate and allowed to cool to room temperature for 40–45 min. After this time, the mixture should be completely tempered. 

**Scheme 1:**
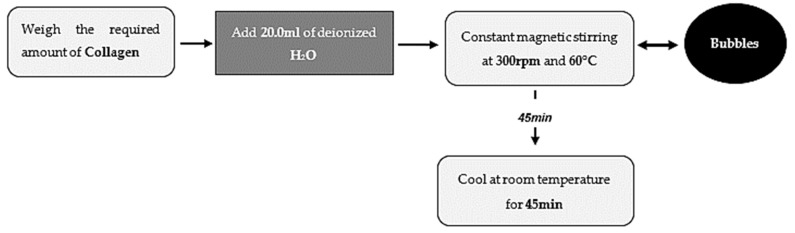
Collagen solution preparation.

The above description is summarized in Scheme 1.

#### 3.2.3. Mono-Layer Membrane Preparation

**Scheme 2:**
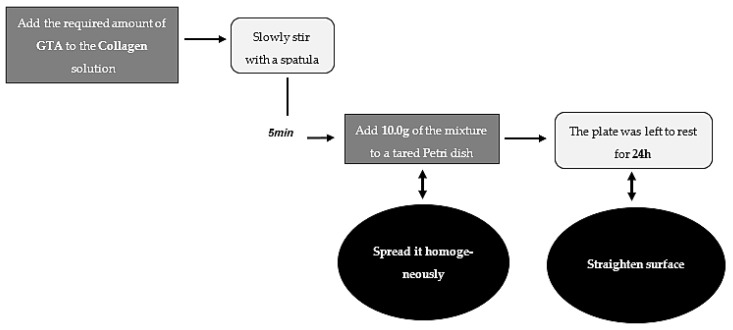
Mono-layered membrane manufacturing process.

Subsequently, the required quantity of GTA was added to the collagen solution on an analytical balance using an automatic pipette. The mixture was then stirred slowly with a spatula for about 5 min, during which a semi-solid formation with a darker and more differentiated color was observed. Just after manual stirring, a Petri dish was tared and 10.0 g of this solution was poured into the Petri dish, ensuring that it was spread evenly over the entire surface of the dish. It was critical to perform this step consecutively as further mixing would cause the solution to coagulate and thus, solidify into an amorphous gelatinous mass. Finally, the plate was left to rest for 24 h on a completely level table to ensure that the membrane was well distributed and there were no irregularities in its thickness. It was important to ensure that every part of the membrane had the same thickness, otherwise the tests would be inconclusive (Scheme 2). 

#### 3.2.4. Preparation of the Tri-Layered Membrane

**Scheme 3:**
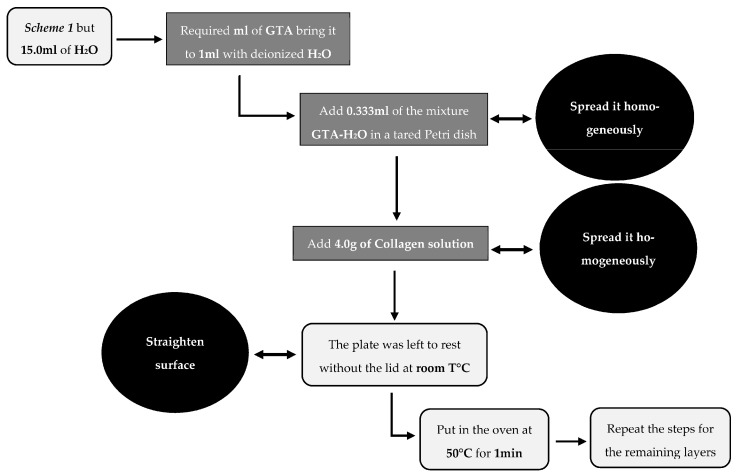
Tri-layered membrane manufacturing process.

The aim was to form the layers one on top of the other and in turn bind them together by binding the unreacted ε-amino groups of the collagen that had not reacted when the membrane was formed. For the creation of a tri-layer membrane, the collagen solution was prepared in exactly the same way, except that instead of 20.0 mL it was 15.0 mL. This is so as to maintain the % *w*/*w* of the formula, since a solution of GTA and collagen is not prepared but rather handled separately. Regarding GTA, the required amount was made up to 1 mL with distilled water so that it would be sufficient to contact the entire collagen surface and at the same time the amount of water would be small so that it would evaporate while each of the layers coagulated. A Petri dish was tared on a balance and 0.333 mL of the GTA-H_2_O solution was added in order to cover all the surface, followed by 4.0 g of the collagen solution. Once the solution was homogeneously distributed on the plate and without covering it with the lid, it was left to stand at room temperature for 6 min and then placed in the oven at 50 °C for 1 min. By this time the membrane should have coagulated, and the water should have evaporated. This was done in exactly the same way for the remaining 2 layers. However, the weight of collagen in the top two layers is 3.0 g, resulting in a final membrane of 10.0 g. The bottom membrane or basement membrane was set at 4.0 g to support the rest (Scheme 3). 

#### 3.2.5. Membrane Curation Process

This process was carried out in the same way for both mono- and tri-layered membranes. It was a process used to finish crosslinking the membrane and increase its strength and structural integrity. It was carried out 4 h after the membrane was manufactured.

The process consisted of a bath with a 10% *w*/*w* solution of GTA for 1 h. During this time, the bath was constantly stirred at 500 rpm to homogenize the solution and distribute it throughout the beaker so that it met the entire surface of the membrane. To prevent the magnet from breaking the membrane when rotating, an aluminum mesh was used as a support and adjusted to the size of the beaker used.

**Scheme 4:**
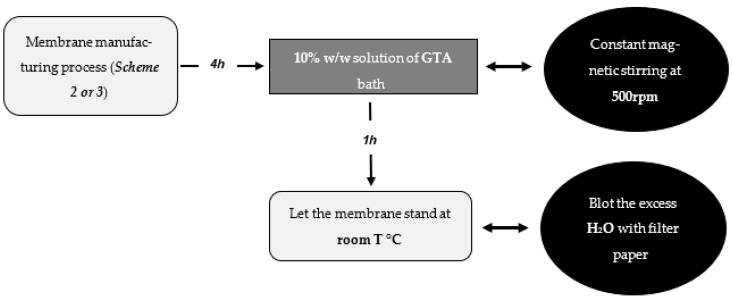
Membrane curation process.

After the bath, the membrane was placed in a Petri dish and left to stand at room temperature for 24 h. After this time, the membrane was observed to have exuded the excess water it had absorbed during the bath and was dried using filter paper. Finally, the membrane was ready for characterization tests (Scheme 4).

### 3.3. Characterization 

The different membranes studied in this work have a specific identification system detailed below with an example:

M3c-C8-G1.2
First position: M = membrane
Second position: 1, 2, 3 = number of layers
Third position: c = cured
Fourth position: C = collagen
Fifth position: 8, 9 or 11 = % (*w*/*w*) collagen
Sixth position: G = Glutaraldehyde
Seventh position: 1.2; 1.3; and 1.5 = % (*w*/*w*) glutaraldehyde

All characterization tests were carried out with mono- and tri-layered membranes, in order to study their behavior and influence on their characteristics.

#### 3.3.1. Rheology Test

This test was carried out using a rheometer AR 2000 ex from TA instruments, (New Castle, DE, USA). It is a very common test used to mechanically characterize materials and to determine their degree of viscoelasticity. 

To obtain the elastic modulus, the membrane, previously die-cut to a diameter of 20 mm, was subjected to an oscillatory movement procedure. The test conditions were a frequency of 1 Hz, strain between 0.01 and 100.00%, a constant temperature of 25 °C and use of a 20 mm striped steel plate. The membrane was placed on the lower plate and the rheometer motor lowered the upper geometry until a normal force of approximately 1.5 N was applied.

#### 3.3.2. Weight Loss Test

This test was carried out using a KERN DBS balance. The moisture content of the sample, whether liquid, solid or semi-solid, can be determined quickly and reliably by thermogravimetric analysis. The duration of the test is determined by the amount of water in the sample, so during the test the time (x) is plotted against the % moisture content (y) and will stop when the moisture values are constant. The lamp reaches a temperature of 100 °C and at the end of the test the weight variation of the sample is obtained.

#### 3.3.3. Swelling Index Assay

This test studies the capacity of a material to absorb a liquid in relation to its dry weight. For this purpose, the phosphate buffer solution (PBS, pH 7.4) previously explained was used. The ability of the material to absorb the PBS was evaluated after a certain period of time and after it had been dried for 24 h in a desiccator with calcium chloride. To make sure that the sample was completely dehydrated, several weighings were carried out until the weight was constant.

**Figure 11:**
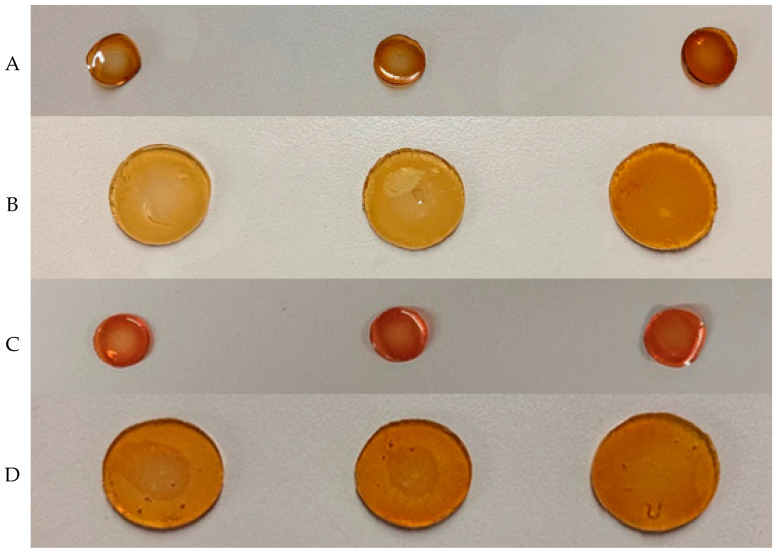
(**A**)—dehydrated mono-layered membranes, from left to right: M1-C8-G1.2; M1-C9-G1.3; and M1-C11-G1.5; (**B**)—fully hydrated monolayer membranes, from left to right: M1-C8-G1.2; M1-C9-G1.3; and M1-C11-G1.5; (**C**)—dehydrated tri-layered membranes, from left to right: M3-C8-G1.2; M3-C9-G1.3; and M3-C11-G1.5; (**D**)—fully hydrated tri-layered membranes, from left to right: M3-C8-G1.2; M3-C9-G1.3; and M3-C11-G1.5.

The test was carried out for both mono-layered membranes and tri-layered membranes (Figure 11). Once completely dry, a final weighing (Wd) was performed, and they were immersed in the PBS solution until the maximum weight was reached. The PBS solution was prepared by dissolving one tablet of phosphate-buffered saline solution, which produces a solution of 0.0100 M phosphate, 0.0027 M potassium chloride and 0.1370 M sodium chloride at pH 7.4 and at 25 °C, in 200 mL of deionized water. 

While membranes were immersed in PBS, the solution was kept under constant agitation at room temperature and the membranes rested on a metal support made of aluminum mesh. At 30 min intervals, the membranes were removed from the beaker and weighed. 

Each time the membrane was weighed, it was blotted with filter paper to remove excess solution from the surface (Ws). The appearance of the membranes when they reach their maximum weight can be seen in Figure 11 as well.

The following equation was used to calculate the swelling index (SI):

$$SI\left(\%\right)=\frac{Ws-Wd}{Wd}\times 100$$

where Ws and Wd are the weights of the swollen and dry samples, respectively.

#### 3.3.4. Permeation Assay

The permeation test was carried out using a Franz cell from Fisher Scientific, S.L. ^®^ (Newington, NH, USA). The capacity of the donor compartment is 30 mL and with a diffusion area of 1.06 cm^2^.

A DermaStamp^®^ was used during the curing process of the membrane in order to create the pores and fix them. This is a device that has micro needles in which the depth can be adjusted to make perforations, in this case it was set at 3 mm to ensure a complete perforation. 

**Figure 12:**
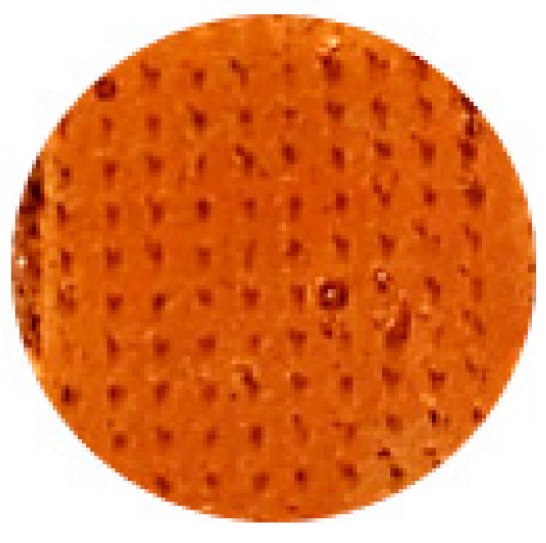
M3-C11-G1.5 fully drilled and activated.

To ensure that all the needles passed through the membrane, they were observed through the lower part of the beaker and through the membrane, as this is translucent, see Figure 12.

After the curation bath with the DermaStamp^®^ drilling the membrane during 1 h at ambient temperature, the membrane was dried with a filter paper to remove excess liquid from the surface and left to stand for 24 h.

#### 3.3.5. Scanning Electron Microscopy

The scanning electron microscope or SEM produces high resolution images from a beam of high-energy electrons that move over the surface of the membrane to generate a variety of signals at the surface. The signals that derive from electron–sample interactions reveal information about the sample including external morphology, porosity, roughness, etc. The sample is gassed with Argon under a vacuum to metallize it, as it is non-conductive and would otherwise be destroyed by the electron beam. A JEOL JSM-5310 electron microscope was used in this study.

## 4. Conclusions

Chemical crosslinked collagen membranes were obtained using GTA as a crosslinking agent. The development of this methodology shows good reproducibility to obtain tri-layered and mono-layered crosslinked scaffolds of collagen. It has been shown that the obtained membranes were sensitive to different properties of human skin such as permeability, absorption, dehydration and elasticity.

In addition, both crosslinking and the use of three-layer structures have been shown to be crucial to increase the elastic modulus of the membrane and approximate the elasticity of human skin. Furthermore, if these layers are crosslinked during the curing process, the elasticity increases much more, especially when the collagen concentration increases (Mx-C11-G1.5). This formulation in question has turned out to be similar in terms of elasticity to human skin in different parts of the body such as the forearm or the abdominal wall (8 kPa–15 kPa).

Regarding the G′ modulus values obtained, the tri-layered membranes turned out to be more elastic, under equal conditions, than the mono-layer membranes. For cured membranes, the results were around 14.0 kPa vs. 9.0 kPa, and for uncured membranes, 11.0 kPa vs. 6.0 kPa.

On the other hand, it has been determined that most of the water present in these collagen membranes was free water. So, just like skin, they suffer processes such as dehydration or hydration. 

Regarding permeation, it has been observed that mechanical pores are required to permeate through the membrane and therefore completely perforate all three layers. This is like the integumentary system that has channels and pores for the exchange of salts or to produce processes such as sweating. It could be interesting to compare an application of cosmetic products with moisturizing claims against a placebo.

Significant differences have been observed with respect to chitosan membranes [17]. On the one hand, chitosan membranes with 85% DDA have a significantly higher G′ modulus than collagen membranes (35–54 kPa), whereas membranes with 76% DDA are much less elastic (Tables S1–S5). On the other hand, collagen membranes have an LVL three times higher (10–12 min vs. 2–3 min) under the same rheological conditions, so it can be ensured that they have greater structural resistance. Furthermore, collagen membranes have a higher absorptive capacity, but chitosan membranes have a higher stability in swelling kinetics. Each of them has its advantages with respect to the objective desired to achieve with them.

Throughout the study carried out, an attempt has been made to highlight one of the possible alternatives that could be the basis for creating skin models and thus be able to develop all the in vitro tests of the cosmetic industry on them in the near future. As discussed, collagen membranes can become similar in certain aspects to human skin, in fact, in terms of elasticity, they have been able to get closer to reality. In addition, if it is desired to study skin from parts of the human body such as the forearm, one could select which membrane is the most suitable for it, depending on the place in the body and the characteristic to evaluate [25].

Finally, these tri-layered membranes manufactured from collagen can be the continuation of a line of research for the creation of other membranes that combine different topical compounds or active ingredients such as elastin or lipid complexes, depending on the objective to be studied. It is only a matter of time and investment before collagen tri-layer membranes can bring their potential and utility to the pharmaceutical and cosmetic industry.

## Figures and Tables

**Table 1:** Real values of the elastic modulus of the skin in different parts of the human body, classified by sex. Reprinted/adapted with permission from Ref. [25]. 2023, Yujia Yang, Liyun Wang, Feng Yan, Xi Xiang, Yuanjiao Tang, Lingyan Zhang, Jibin Liu, Li Qiu.

Place in the Body	Sex	Mean Elastic Modulus (kPa)
Finger	Men	31.8 ± 10.0
	Women	28.7 ± 8.3
Forearm	Men	16.1 ± 5.5
	Women	13.4 ± 5.8
Chest wall	Men	19.9 ± 6.1
	Women	15.7 ± 4.3
Abdominal wall	Men	10.4 ± 3.3
	Women	8.6 ± 2.5

**Table 2:** Average weights and thicknesses for the mono- and tri-layered membranes of the three formulations.

Membrane ID	Mean Membrane Weight (g) ± SD	Mean Membrane Thickness (mm) ± SD
M1-C8-G1.2	0.451 ± 0.01	0.832 ± 0.12
M1-C9-G1.3	0.455 ± 0.05	0.865 ± 0.055
M1-C11-G1.5	0.430 ± 0.02	0.980 ± 0.08
M3-C8-G1.2	0.519 ± 0.15	1.2 ± 0.25
M3-C9-G1.3	0.524 ± 0.40	1.08 ± 0.15
M3-C11-G1.5	0.524 ± 0.10	1.16 ± 0.1

**Table 10:** Final values obtained during the test for comparison with the theoretical amount of the formulations. Formulations classified by color as in Table 3.

Membrane ID	% Humidity (Water Loss) at 60 min
M1-C8-G1.2	91.14
M3-C8-G1.2	89.89
M1-C9-G1.3	89.77
M3-C9-G1.3	87.82
M1-C11-G1.5	88.22
M3-C11-G1.5	86.03

**Table 11:** Initial and final weights of each of the membranes in the swelling index test. Formulations classified by color as in Table 3.

	M1-C8-G1.2	M3-C8-G1.2	M1-C9-G1.3	M3-C9-G1.3	M1-C11-G1.5	M3-C11-G1.5
Initial Weight (g)	0.451	0.529	0.425	0.524	0.450	0.514
Dry Weight (g)	0.044	0.058	0.053	0.066	0.079	0.081
Swollen Weight (g)	0.268	0.289	0.290	0.354	0.420	0.449
Recovered Weight (%)	59.4	54.6	68.2	67.6	93.3	87.4

**Table 12:** Experimental DOEs. Δ refers to a change or difference.

DOE	Δ Collagen % (*w*/*w*)	Δ GTA % (*w*/*w*)	Drying Time (Days)	Best Average of G′ Modulus (Pa)
1	6.0–8.0	1.0–1.2	1–2	2639.70
2	7.0–9.0	1.1–1.3	1–2	5685.29
3	9.0–11.0	1.3–1.5	1–2	5962.14

**Table 13:** Final formulations.

Formulations	Collagen % (*w*/*w*)	GTA % (*w*/*w*)	Drying Time (Days)
Mx-C8-G1.2	8.0	1.2	1
Mx-C9-G1.3	9.0	1.3	1
Mx-C11-G1.5	11.0	1.5	1

## Data Availability

No new data were created or analyzed in this study. Data sharing is not applicable to this article.

## Supplementary Materials

The supporting information can be downloaded at: https://www.mdpi.com/article/10.3390/ijms241713443/s1.
