# Supplementary material for: Creation of Chemically Tri-Layered Collagen Crosslinked Membranes and Their Comparison with Ionically Tri-Layered Chitosan Crosslinked Membranes to Study Human Skin Properties
Source: Int J Mol Sci. 2023 Aug 30;24(17):13443. doi: 10.3390/ijms241713443 (PMC10487804; doi:10.3390/ijms241713443)
Supplement: Supplementary file 1 [file ijms-24-13443-s001.zip › ijms-2570967-Supplementary Material.pdf]

Supporting Information

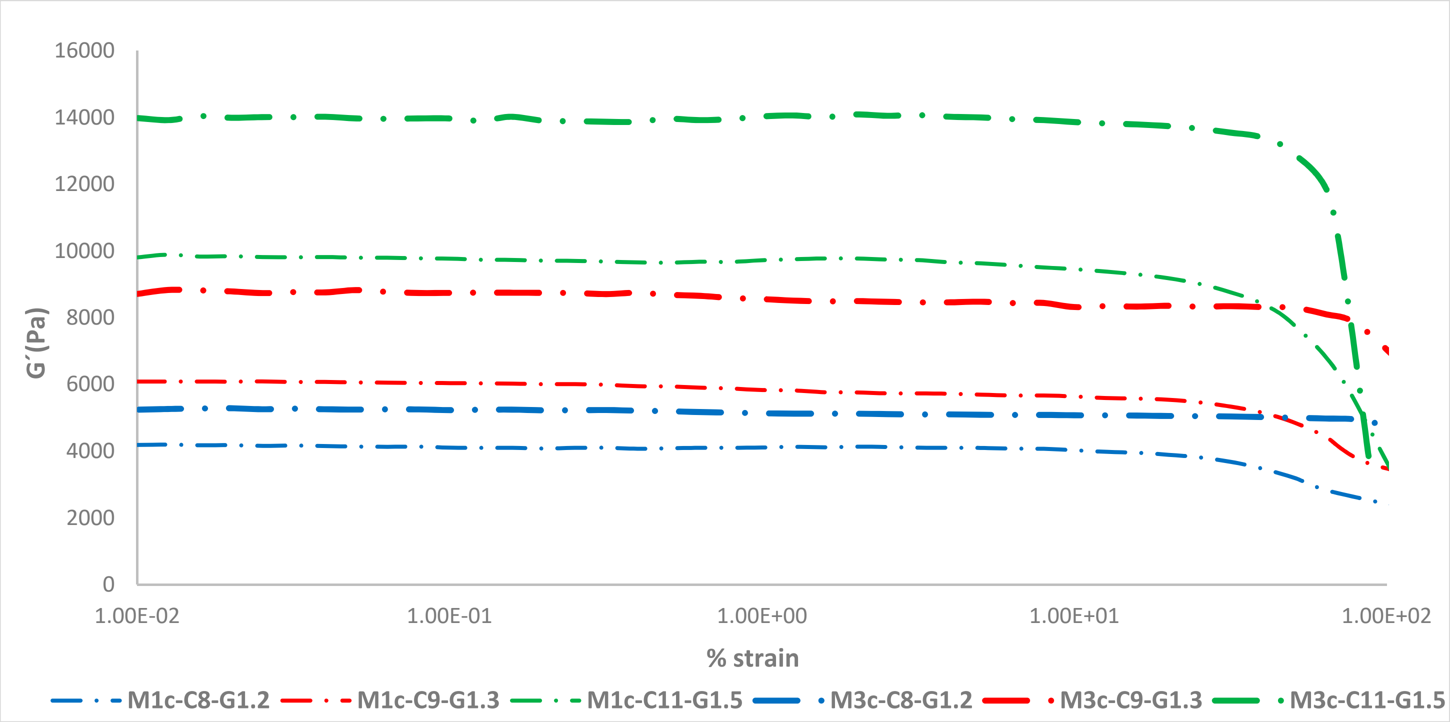

Figure S1. Representation of the effect of curing on the elastic modulus of cured mono-layered and cured tri-layered membranes.

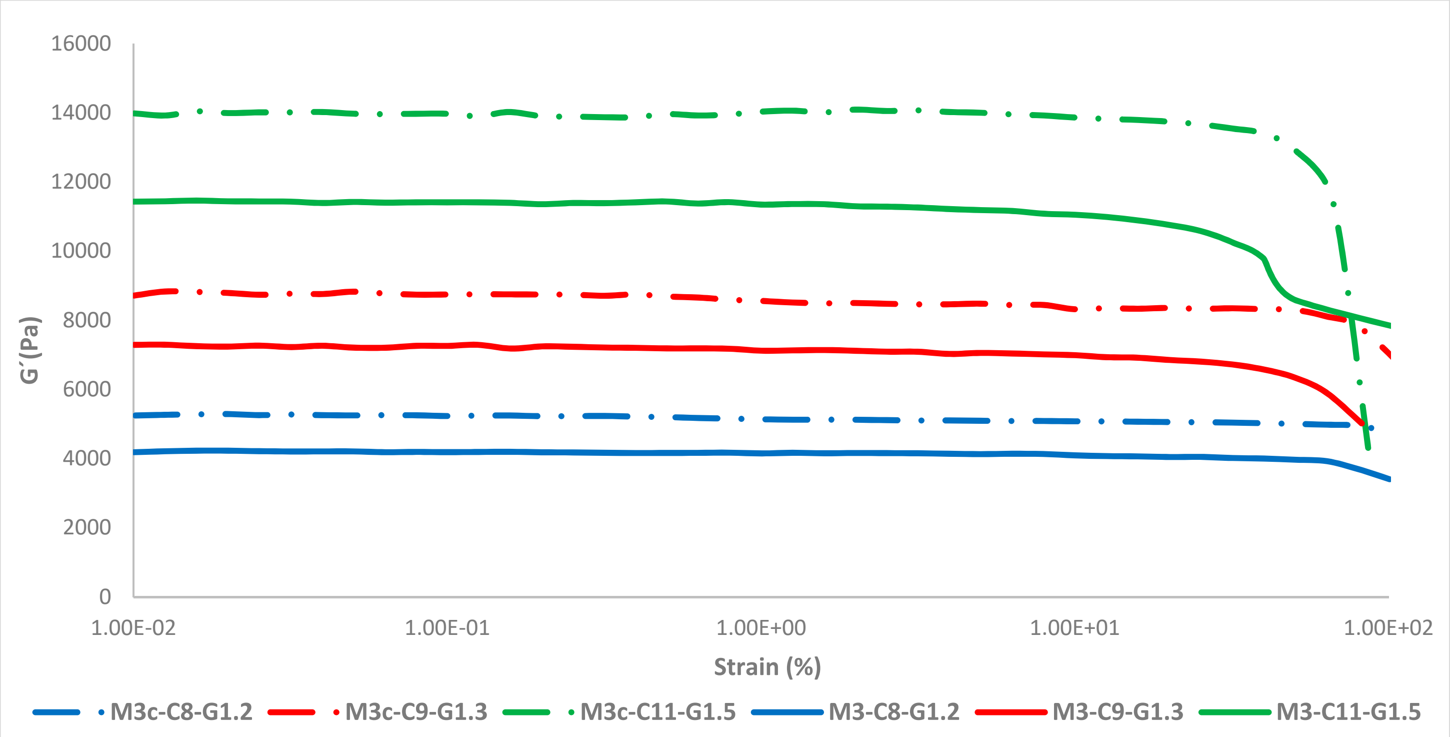

Figure S2. Representation of the curation effect on the elastic modulus of cured and non-cured tri-layered membranes.

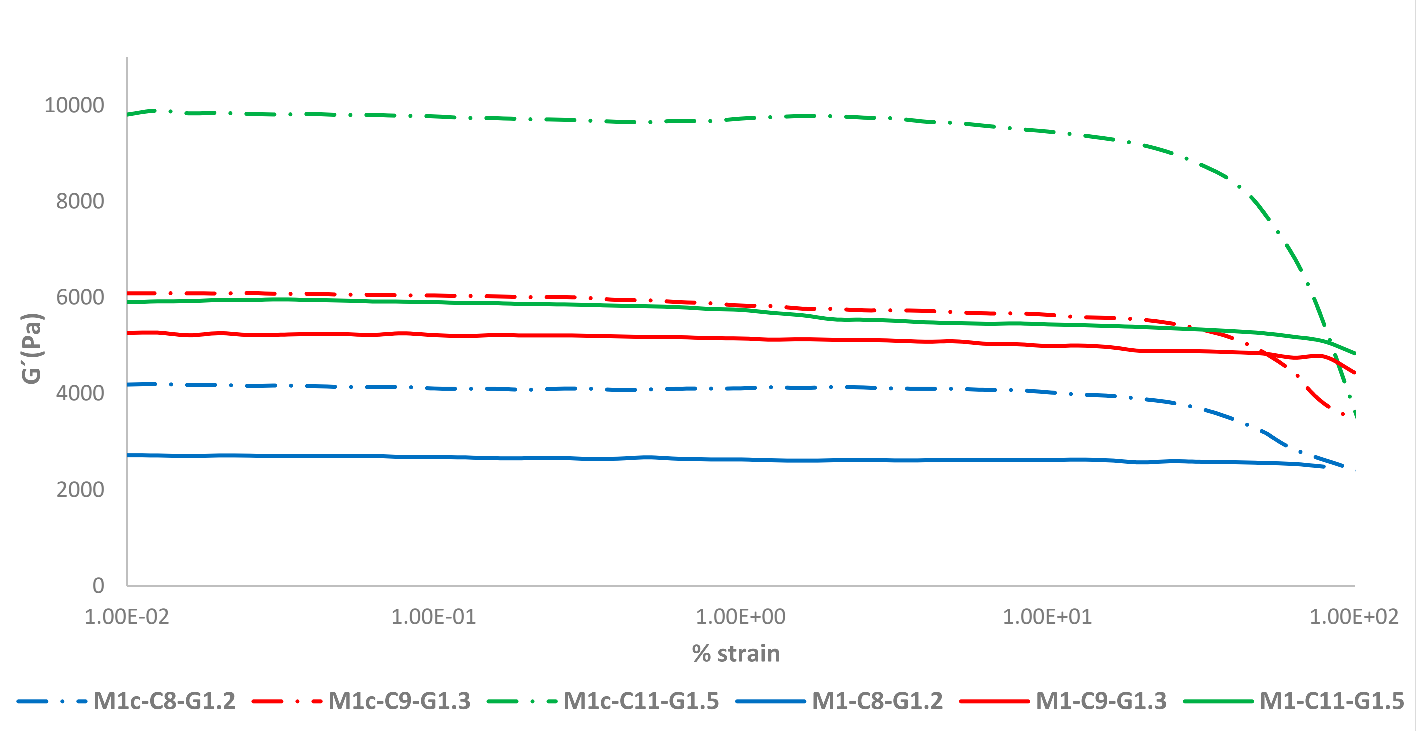

Figure S3. Representation of the curation effect on the elastic modulus of cured and non-cured mono-layered membranes.

Table S1. Special identification system for chitosan membranes [17].

Position	Variable data
First	**M** = membrane
Second	**3**, **2**, **1** = number of layers
Third	**B** = base membrane or **A** = activated membrane
Fourth	**C** = Chitosan
Fifth	**85** or **76** = %DDA
Sixth	**A** or **H** = Aldrich or HMC + supplier
Seventh	**T** from TPP crosslinker
Eighth	**X** or **0** = crosslinked or non-crosslinked

Table S2. Swelling comparative table of results with chitosan membranes [17].

Chitosan membranes ID	Mean Maximum Swell% ± SD	Mean Maximum Swell% ± SD	Collagen Membranes ID
M3B-C85A-TX	107.0 ± 7.4	337.0 ± 5.5	M3-C8-G1.2
M3A-C85A-TX	114.0 ± 6.2	341.7 ± 4.9	M3-C9-G1.3
M3B-C85H-TX	114.0 ± 5.2	352.9 ± 4.1	M3-C11-G1.5
M3A-C85H-TX	115.0 ± 5.3	426.4 ± 18.3	M1-C8-G1.2
M3B-C76A-TX	256.0 ± 63.6	382.7 ± 15.7	M1-C9-G1.3
M3A-C76A-TX	386.0 ± 122.3	343.4 ± 11.1	M1-C11-G1.5

Table S3. Comparative table of results of the rheometric test with non-crosslinked layers in tri-layered chitosan membranes [17].

Chitosan membranes ID	Mean G' (Pa) ± SD	Mean G' (Pa) ± SD	Collagen Membranes ID
M3B-C76A-T0	2141.58 ± 198.96	4114.66 ± 559.36	M3-C8-G1.2
M3A-C76A-T0	2587.29 ± 608.81	7014.64 ± 196.32	M3-C9-G1.3
M3B-C85A-T0	31,332.35 ± 7033.19	11,012.53 ± 100.60	M3-C11-G1.5
M3A-C85A-T0	37,512.59 ± 4967.79		

Table S4. Comparative table of results of the rheometric test with cured tri-layered chitosan membranes [17].

Chitosan membranes ID	Mean G' (Pa) ± SD	Mean G' (Pa) ± SD	Collagen Membranes ID
M3B-C76A-TX	3559.46 ± 968.97	5246.19 ± 202.30	M3c-C8-G1.2
M3A-C76A-TX	5402.78 ± 1,096.34	8607.78 ± 95.96	M3c-C9-G1.3
M3B-C85H-TX	34,378.43 ± 2,702.69	13,677.95 ± 281.44	M3c-C11-G1.5
M3A-C85H-TX	35,538.68 ± 9,561.26		
M3B-C85A-TX	53,633.68 ± 2,413.52		
M3A-C85A-TX	45,997.65 ± 5,515.39		

Table S5. Comparative table of results of the rheometric test with mono-layered chitosan membranes [17].

Chitosan membranes ID	Mean G' (Pa) ± SD	Mean G' (Pa) ± SD	Collagen Membranes ID
M1A-C76A	874.70 ± 161.95	2638.95 ± 216.54	M1-C8-G1.2
M1B-C76A	996.11 ± 6.48	3946.16 ± 373.22	M1c-C8-G1.2
M1A-C85A	20,299.41 ± 1,053.55	5685.29 ± 284.25	M1-C9-G1.3
M1B-C85A	21,199.80 ± 5,058.00	5758.55 ± 157.37	M1c-C9-G1.3
		5695.77 ± 92.85	M1-C11-G1.5
		9329.93 ± 417.36	M1c-C11-G1.5

Table S6. DOE 1 variable data.

Collagen % (w/w)	GTA % (w/w)	Dry time (days)	
8.0	1.2	2	**+**
6.0	1.0	1	**-**

Table S7. Results of DOE 1 G´ modulus.

Membrane	GTA	Collagen	t dry	GTA x Collagen	GTA x t dry	Collagen x t dry	Mean of G´ (Pa)
1	+	-	-	-	-	+	1698.00
2	+	+	-	+	-	-	2639.70
3	+	-	+	-	+	-	1331.17
4	+	+	+	+	+	+	1822.29
5	-	-	-	+	+	+	1470.68
6	-	+	-	-	+	-	2546.68
7	-	-	+	+	-	-	1175.80
8	-	+	+	-	-	+	2155.98

Table S8. Effects and interactions of the variables regarding module G' of the DOE 1.

Effects	Results	Interactions	Results
**GTA**	35.25	**GTA x Collagen**	-155.75
**Collagen**	872.25	**GTA x t dry**	284.25
**t dry**	-58.75	**Collagen x t dry**	272.25
		**GTA x Collagen x t dry**	320.25

Table S9. DOE 2 variable data.

Collagen % (w/w)	GTA % (w/w)	Dry time (days)	
9.0	1.3	2	+
7.0	1.1	1	-

Table S10. Results of DOE 2 G´ modulus.

Membrane	GTA	Collagen	t dry	GTA x Collagen	GTA x t dry	Collagen x t dry	Mean of G´ (Pa)
1	+	-	-	-	-	+	2746.90
2	+	+	-	+	-	-	5685.29
3	+	-	+	-	+	-	3484.38
4	+	+	+	+	+	+	5097.67
5	-	-	-	+	+	+	2273.37
6	-	+	-	-	+	-	3434.05
7	-	-	+	+	-	-	3487.08
8	-	+	+	-	-	+	4145.46

Table S11. Effects and interactions of the variables regarding module G' of the DOE 2.

Effects	Results	Interactions	Results
**GTA**	918.75	**GTA x Collagen**	683.25
**Collagen**	1,592.75	**GTA x t dry**	-443.75
**t dry**	518.75	**Collagen x t dry**	-456.75
		**GTA x Collagen x t dry**	-205.25

Table S12. DOE 3 variable data.

Collagen % (w/w)	GTA % (w/w)	Dry time (days)	
11.0	1.3	2	**+**
9.0	1.5	1	**-**

Table S13. Results of DOE 3 G´ modulus.

Membrane	GTA	Collagen	t dry	GTA x Collagen	GTA x t dry	Collagen x t dry	Mean of G´ (Pa)
1	+	-	-	-	-	+	3967.74
2	+	+	-	+	-	-	5962.14
3	+	-	+	-	+	-	2568.91
4	+	+	+	+	+	+	4003.29
5	-	-	-	+	+	+	2418.80
6	-	+	-	-	+	-	5707.54
7	-	-	+	+	-	-	Null
8	-	+	+	-	-	+	4282.15

Table S14. Effects and interactions of the variables regarding module G' of the DOE 3.

Effects	Results	Interactions	Results
**GTA**	948.5	**GTA x Collagen**	-1111.0
**Collagen**	2675.0	**GTA x t dry**	1026.0
**t dry**	-896.0	**Collagen x t dry**	1012.5
		**GTA x Collagen x t dry**	516.5
